# First line anti-tuberculosis induced hepatotoxicity: incidence and risk factors

**DOI:** 10.11604/pamj.2016.25.167.10060

**Published:** 2016-11-16

**Authors:** Omaima El Bouazzi, Sanaa Hammi, Jamal Eddine Bourkadi, Amina Tebaa, Driss Soussi Tanani, Rachida Soulaymani-Bencheikh, Narjis Badrane, Rachid Bengueddour

**Affiliations:** 1 Centre Anti Poison et de Pharmacovigilance du Maroc, Rabat, Maroc; 2 Faculté des Sciences, Universités Ibn Tofail, Kénitra, Maroc; 3 Faculté de Médecine et de Pharmacie, Université Abd El Malek Essadi, Tanger, Maroc; 4 Hôpital Moulay Youssef, Rabat, Maroc; 5 Faculté de Médecine et de Pharmacie, Université Mohammed V, Rabat, Maroc

**Keywords:** Tuberculosis, hepatotoxicity, therapeutic drug monitoring, risk factor

## Abstract

In our days, tuberculosis, whet ever its localization, became a curable disease. The cornerstone is a 6 month course of isoniazid, rifampicine and pyrazinamide. All of the three first line antituberculosis drugs may induce hepatic damage which may have negative consequences for treatment outcome. Several risk factors were associated with the development of antituberculosis- drug-induced hepatotoxicity (ATDH). A retrospective study was conducted from July 2014 to March 2015 regarding all therapeutic drug-monitoring requests sent to the Laboratory of Poison Control and Pharmacovigilance Centre of Morocco. 142 patients diagnosed with active tuberculosis were included in study. Plasma peak levels of isoniazid, rifampicin and pyrazinamide were analyzed in plasma samples after 2 to 3 hours of administration of anti-tuberculosis treatment. Logistic regression was used to identify the ATDH risk factors. The incidence of ATDH was found 24.6% (35 patients out of 142). Intergroup differences in the plasma levels were statistically significant for isoniazid (p=0.036). ATDH was found to be associated with combined form of anti-TB drugs (p=0.002, COR=13.1, AOR= 13.5) and plasma concentration of INH superior to 2mg/l (p=0.045, COR=1.3, AOR= 1.4).age, gender, alcohol intake and smoking status were not significantly associated with ATDH. The finding of 24.6% incidence of hepatotoxicity is extremely high. Many factors can be associated with the development of ATDH such as genetic factors, combined forms of treatment and plasma peak levels.

## Introduction

Tuberculosis (TB) remains a global major health Problem, especially in developing countries. Since 1993, World Health Organization (WHO) has declared TB as a public health emergency [1]. In 2014, there was an estimated 9.6 million new cases reported to WHO and 1.5 deaths around the world [2]. In Morocco, TB remains the leading cause of serious illness with an estimated incidence of 106 (97-105) per 100000 in 2014 [3]. Recommended standard treatment includes a combination of isoniazid (INH), rifampicin (RIP), pyrazinamide (PZA) and éthambutol (EMB) for 6-9 months [4–6]. Although this treatment regimen has been highly effective, treatment-related adverse effects including hepatotoxicity, skin reactions, gastrointestinal and neurological disorders account for significant morbidity leading to reduced effectiveness of therapy [1, 7, 8]. Hepatotoxicity is the most important and serious one [7, 9, 10]. It may result from the direct toxicity of the primary compound, a metabolite, or from an immunologically mediated response [11]. Antituberculosis drug-induced hepatotoxicity (ATDH) causes substantial morbidity and mortality and diminishes treatment effectiveness. There are many factors that contribute to the development of ATDH, which are advanced age, female sex, slow acetylor status, malnutrition, HIV infection and preexistant liver disease [1, 7, 10, 11]. Therapeutic drug monitoring (TDM) allows the clinician to make informed decisions in cases to avoid treatment failures or prevent the occurrence of adverse events. The relationship between ATDH and plasma drug levels has not been demonstrated. There are few reports on the correlation between basal plasma drugs levels and ATDH. The objective of this study was to identify ATDH specific risk factors in moroccan population and to evaluate the association between the basal plasma levels and the development of ATDH.

## Methods

### Study design and patients

Demographic and clinical data as well as concentration measurements of anti-TB drugs were collected prospectively from the TDM service at the laboratory of the Poison Control and Pharmacovigilance Centre of Morocco (CAPM-LAB), from 07/01/2014 to 03/31/2015 (9 months). The data included TDM requests of patients with pulmonary or extra pulmonary TB receiving anti-TB drugs such as INH, RIF and PZA according to their body weight as part of their treatment regime. Treatment was planned according to the National TB Control Program (PNLAT) recommendations. The inclusion criteria were: patients aged 15 years or older whose human immunodeficiency virus, hepatitis B and hepatitis C serological tests; serum aminotransferases (AST, ALT) and bilirubin levels were available; who were treated with isoniazid, rifampicin and pyrazinamide during the hospitalization. We have excluded the patients with history of cirrhosis of the liver or chronic hepatitis from our study.

### Characteristics of ATDH

**Figure 1:**
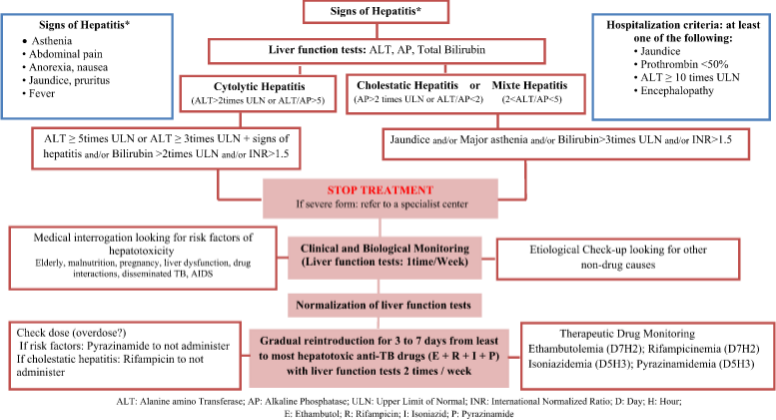
Management of Anti-TB induced hepatotoxicity in Morocco

Drug induced hepatitis was defined as normalization of liver enzyme levels and resolution of signs and symptoms of hepatotoxicity after withdrawal of all anti-TB drugs [7]. In Morocco, The management of Anti-TB drugs induced hepatotoxicity have been established by the Moroccan technical committee of Anti-TB drugs pharmacovigilance, it’s summarized in the Figure 1.

### Anti-TB drugs therapeutic drug monitoring in the CAPM-LAB

The sampling is performed after 2 weeks of the beginning of the treatment because of the expected steady-state in the pharmacokinetics of when of the first line TB drugs. A blood sample was taken 2 to 3 hours after anti-TB drug intake on an empty stomach to estimate the peak plasma concentration (Table 1). Plasma was then separated by centrifugation and frozen to -20°C. High performance liquid chromatography (HPLC) results were available within 24 hours of receipt of specimen and reported in reference to the expected µg/mL range (Table 1). These ranges represent the normal concentrations that can be expected after the standard doses of TB drugs.

**Table 1:** Pharmacokinetic parameters of anti-TB drugs

Drug	Expected time of C max (Hours)	Expected C max range (µg/mL)
Isoniazid	3	1-2
Rifampicin	2	8-24
Pyrazinamid	2	30-50

### Data analysis

The results are shown as averages, median or frequency of patients with a given characteristic. The statistical tests used in this analysis are: Chi-square test, fisher’s exact test and student’s t test. The chosen significance level was p <0.05. Univariate logistic regression was performed on all clinical and baseline TDM parameters. Multivariate logistic regression was done to all significant (p <0.20) parameters to calculate adjusted odds ratio.

### Risk factors

Studied risk factors were: age>35 years, female sex, smoking status, alcoholism, cannabis consumption, HIV positive, malnutrition, renal failure, form of TB, hepatitis B virus positive, combined form of anti-TB drugs and plasma concentration of INH, RIF and PZA [1, 7, 10–14].

## Results

During the study period, 142 active TB cases were on treatment. The mean age for patients in the study was 42.6±17.7 years and 59 (41.5%) were women with sex ratio (M/F) of 1.4. The most representative age groups were adults (83.8%), old people (13.4%) and adolescents (2.8%). The active pulmonary TB diagnosis was bacteriologically and clinically confirmed in 116 cases (81.7%). HIV and HBV co-infection were detected respectively in 11(7.7%) and 5(3.5%) patients. 15(10.6%) had history of cannabis use, 14(9.9%) of alcoholism and 56(39.4%) were smokers. Patients’ characteristics results are summarized in Table 2. 444 TDM assays were performed including 198 (44.6%) analyzes of INH, 179 (40.3%) analyzes of RIF and 67 (15.1%) analyzes of PZA.

**Table 2:** Clinical characteristics of study patients (N=142)

Characteristic	Findings
**Female sex+,sex ratio (M/F)**	59 (41.5), 1.4
**Age, years+++**	52.1±17.9
**Body weight, Kg+++**	52.5±12.9
**Malnutrition+**	47(33.1)
**Form of TB +**	
Pulmonary	116 (81.7)
Extrapulmonary	19 (13.4)
Miliary/ disseminated	7(4.9)
**History of TB+**	21(14.8)
Comorbidities+	
Renal failure	10 (7)
Diabetes	12 (8.5)
HIV coinfection	11(7.7)
Hepatitis B coinfection	05(3.5)
Hepatitis C coinfection	00 (00)
**Smoking status+**	56(39.4)
**Alcohol consumption+**	14(9.9)
**Cannabis consumption+**	15(10.6)
**Anti-TB treatment+**	
INH+RIF+PZA+ETB	112(78.9)
INH+RIF	21(14.8)
INH	7(4.9)
RIF	2(1.4)
**Anti-TB treatment adverse reactions+**	
Liver and biliary system disorders	35(24.6)
Body as a whole- general disorders	9(6.3%)
Gastro-intestinal system disorders	15(10.6%)
Psychiatric disorders	2(1.4%)
Vascular, bleeding and clotting disorders	3(2.1%)
Metabolic and nutritional disorders	1(0.7%)

+ Number (%)

++ median range

+++ mean ± SD.

35 (24.6%) of all patients developed hepatotoxicity. Apart hepatotoxicity, patients developed other adverse effects on TB treatment: body as a whole- general disorders (6.3), gastro-intestinal system disorders (10.6%), psychiatric disorders (1.4%), vascular, bleeding and clotting disorders (2.1%) and metabolic and nutritional disorders (0.7%) (Table 2).

ATDH was detected in 35(24.6%) patients. The mean age of those patients was 48.1±19 years and 18 (51.4%) were women. ATDH was cytolytic in 65.7% of cases, cholestatic in 11.4% and mixed in 22.9% of cases. There were 60 % patients who developed late ATDH. The median latent period for developing ATDH was 19 [10-25] days. 21 (60%) patients were symptomatic with asthenia (28.6%), nausea (31.4%), abdominal pain (22.9%), jaundice (22.9%), pruritus (17.1%), fever (20%), and anorexia (28.6%) (Table 3).

**Table 3:** Baseline characteristics of patients showing ATDH (N=35)

Characteristics	Findings
**Age, years+++**	48.1±19
**Female sex+,sex ratio (M/F)**	18(51.4),0.9
**Classification of ATDH+**	
Hepatocellular hepatitis	23(65.7)
Cholestatic hepatitis	4(11.4)
Mixed hepatitis	8(22.9)
**Time of development of the ATDH++ (days)**	19(10-25)
Early * (<15 days)	14(40)
Delayed* (>15 days)	21(60)
**Normalization of liver function tests (days)**	18(10-30)
**Symptoms of hepatotoxicity+**	
Asthenia	10(28.6)
Nausea	11(31.4)
Abdominal pain	8(22.9)
Jaundice	8(22.9)
Pruritus	6(17.1)
Fever	7(20)
Anorexia	10(28.6)
**Duration of treatment discontinuation++(day)**	18(11-30)
**Doses (mg/kg/day)**	
INH	4.7(4.4-5.3)
RIF	8.2(7.5-10)
PZA	25.4(23.5-28.6)

+ No (%)

++ median range

+++ mean ± SD

After the confirmation of ATDH, all anti-TB drugs were stopped until normalization of liver function tests (18 (11-30) days). Then there were re-administered, with permanent control of the patient´s condition. When we compared the plasma anti-TB drugs concentration between the two groups (with and without ATDH), no significant differences in the RIF and PZA plasma levels were observed (respectively p= 0.885 and p= 0.309). There was a significant difference between the two groups in the levels of plasma concentrations of INH with p= 0.036 (Table 4).

**Table 4:** Basal plasma concentration of INH, RIF, PZA

Drugs	Patients with hepatotoxicity		Patients without hepatotoxicity		*P* value
	n	Median [range]	n	Median [range]	
Concentration of INH (µg/mL)	30	2(1-4.3)	103	1.3(0.9-2.3)	**0.036**
Concentration of RIF (µg/mL)	24	4.8(1.9-7)	98	4.8(2.9-6.7)	0.885
Concentration of PZA (µg/mL)	5	38.5(33.8-48.5)	50	34.9(24.4-42.3)	0.309

With cox regression model, all variables with p≤0.20 were analyzed (Female sex, cannabis consumption, HIV, pulmonary form, combined form of anti-TB drugs, plasma concentration of INH superior to 2mg/l). The variables associated to the development of ATDH were: combined form of anti-TB drugs and plasma concentration of INH superior to 2mg/l (Table 5).

**Table 5:** Logistic regression analysis of variables associated with ATDH

Variables	*p* value	COR(95% IC)	*p* value	AOR (95% IC)
Age > 35 years	0.23	1.59 (0.73-3.45)	-	-
Female sex	0.17	0.58 (0.27-1.26)	0.33	0.64(0.26-1.57)
Smoking status	0.63	0.82 (0.38-1.79)	-	-
Alcohol consumption	0.76	1.22 (0.32-4.65)	-	-
Cannabis consumption	0.12	5.11 (0.64-40.41)	0.28	3.28(0.37-28.73)
HIV coinfection	0.10	0.35 (0.10-1.25)	0.93	0.93(0.18-4.68)
Malnutrition	0.51	1.32 (0.57-3.044)	-	-
Renal failure	0.68	0.74 (0.18-3.05)	-	-
Pulmonary form	0.12	3.83 (0.14-4.78)	0.50	1.94(0.27-13.71)
Hepatitis B coinfection	0.42	0.47 (0.07-2.97)	-	-
Combined form of drugs	**0.002**	13.12 (2.58-66.70)	**0.003**	13.53(2.37-77.07)
Plasma concentration of isoniazid > 2 mg / l	**0.045**	1.29 (1.01-1.67)	**0.04**	1.45(1.18-3.11)
Plasma concentration of rifampicin>24 mg / l	0.99	-	-	-
Plasma concentration of pyrazinamid > 50 mg / l	0.28	0.95 (0.87-1.03)	-	-

## Discussion

Hepatotoxicity is the most common adverse reaction of anti-TB treatment that leads to interruption of therapy. The exact mechanism of ATDH is not well defined but is due to toxic metabolites. The variation in the incidence of ATDH worldwide depends on several factors: study design, investigors definition of ATDH, population studied and indiscriminate drugs. Studies reported in several countries are shown in the Table 6.

**Table 6:** Incidence and risk factors for ATDH

Year of study	Proportion of ATDH (%)	risk factors	Population	References
2015	24.6	Combined form of anti-TB drugs plasma concentration of INH superior to 2mg/l	Morocco	Our study
2016	18.2	No significant risk factors	Nigeria	[4]
2015	8	Alcoholism	Ethiopia	[11]
2007	19.7	Alcoholism paracetamol low serum cholesterol	Pakistan	[12]
2005	2.6	Alcoholism Hepatitis B virus other hepatotoxic drugs	Spain, SA	[15]
2003	3	Advanced age female sex HIV	As, CA and SA, Af, NA	[16]
2004	3.4	Female sex	Dutch	[17]
2005	27.7	No significant risk factors	Iran	[18]
2002	16.1	Advanced age	India	[19]

Orientals are reported to have the highest rates, especially Iran (27.7%) and Pakistan (19.7%). However other studies reported in developed countries showed low incidences (USA, Spain and Dutch). The analysis of our study showed the incidence of ATDH to be 24.6% with 40% of the events in the first 15 days of treatment. This incidence is almost similar to previous reports of study in Iran and Pakistan. The incidence of ATDH is much higher in studies from developing countries compared with developed countries. Several factors can be at the origin of this difference as: Malnutrition, chronic co-infections, ethnic factors or genetic predisposition.

Several genetic factors influence predisposition to ATDH especially for INH [20]. The risk genotype for ATDH is the N-acetyltransferase 2 (NAT 2) slow acetylor [7]. Recent studies have demonstrated that this genetic variation may be associated with the risk of ATDH. A study done in Morocco reported that the most prevalent genotypes of NAT 2 gene in Moroccan are those which encode slow phenotype (72.39%), leading to a high risk of ATDH [20]. This genetic background can explain the high incidence of ATDH in our population.

The median time to the development of ATDH was 19(10-25) days. This finding differs from those other studies, which reported a median of 28 (14-60) days [4]. This difference is caused by the fact that we have not the evolution of all cases and the shorter period of hospitalization.

The question that arises: why we have an incidence raised in our population and which are factors in cause? And why only some patients develop ATDH? Several authors searched for genetic, environmental and demographic factors. Some studies found as risk factor for ATDH, older age (>50 years), female sex, malnutrition, HIV, HBV co-infection and alcohol intake. However, our study, did not find an association.

In the present study, the development of ATDH was associated with two risk factors. The first one is the taking of the combined forms of anti-TB drugs. The combined use of INH and RIF has been associated with an increased risk of ATDH. RIF induces INH hydrolase, increasing hydrazine production [7]. Other authors consider that monotherapy is preferable for the treatment of latent TB [1]. The second risk factor is in connection with the plasma levels of INH. However, in this study we proved that the RIF and PZA plasma levels are not related to ATDH development. These results confirmed that the slow acetylor status of INH is a risk factor for ATDH. Globally, INH is known for its hepatotoxicity and the plasma concentration can have a role in development of ATDH. Ina Joeng et al (2014) [9] retrospectively analyzed 12 cases and found a correlation between development of ATDH and plasma concentrations levels of INH.

## Conclusion

The incidence of ATDH is high in our population which causes many problems as drug resistance and therapeutic failure. We should take into account this high incidence of ATDH in our population probably caused by the combined form of anti-TB drugs and slow acetylors status of majority of Moroccans. It is also very important to study the molecular mechanisms of ATDH and predisposing risk factors.

### What is known about this topic

- Hepatotoxicity is one of the most frequent adverse events induced by anti-tuberculosis drugs;
- Therapeutic drug monitoring (TDM) is useful for optimization of clinical care, through enabling maximum drug efficacy and minimum drug toxicity.

### What this study adds

- This is the first study of the incidence and risk factors of antituberculosis drugs induced hepatotoxicity in Moroccan population knowing that tuberculosis is the one of the major health problem in Morocco;
- This is the only study of the therapeutic drug monitoring of antituberculosis first-line in Morocco realized in the reference Laboratory of Pharmacology and Toxicology in Morocco.
